# Retrospective analysis of 11 patients with ipsilateral hemiplegia caused by acute cerebral infarction

**DOI:** 10.3389/fneur.2025.1653934

**Published:** 2025-10-27

**Authors:** Lixian Huang, Hongyan Li, Xiaoying Hou, Xiaoyuan Guo, Yihuai Zou

**Affiliations:** Dongzhimen Hospital, Beijing University of Chinese Medicine, Beijing, China

**Keywords:** cerebral infarction, ipsilateral limb weakness, ipsilateral hemiparesis, pyramidal decussation, magnetic resonance imaging

## Abstract

**Objective:**

To summarize the clinical and imaging characteristics of patients with ipsilateral hemiparesis caused by acute cerebral infarction, and to explore possible pathophysiological mechanisms.

**Methods:**

This is a retrospective case series study. Clinical data of 11 patients with ipsilateral hemiparesis caused by acute cerebral infarction admitted to the Neurology Department, Dongzhimen Hospital, Beijing University of Traditional Chinese Medicine from January 1, 2021 to March 30, 2025 were collected. Descriptive analysis was conducted on the patients’ neurological symptoms and signs, magnetic resonance imaging (MRI), blood tests, and other examination results.

**Results:**

Among the 11 patients with acute cerebral infarction, the onset time was between 6 and 48 h. Nine patients did not undergo thrombolysis, two patients underwent thrombolysis, five patients had left hemiparesis, and six patients had right hemiparesis. There were 6 cases of hemiparesis with muscle strength level 4, 3 cases with muscle strength level 5^−^, 1 case with proximal muscle strength level 2 and distal muscle strength level 4, and 1 case with upper limb muscle strength level 4 and lower limb muscle strength level 3^−^. In terms of combining other neurological symptoms, there were 6 cases of speech impairment, 2 cases of hemihypesthesia, 3 cases of facial paralysi, and 4 cases without neurological symptoms. There were 10 cases of anterior circulation and 1 case of posterior circulation, involving infarcted areas such as thalamus, basal ganglia, corona radiata (adjacent to or centrum semiovale), frontal lobe, cerebral peduncle of midbrain, and responsible blood vessels involving involving large artery disease in 7 cases and perforating arteries in 4 cases. Based on the Chinese Ischemic Stroke Subclassification (CISS), the main etiological types are atherosclerosis, perforating artery disease, and other causes (cerebrovascular malformation).

## 1 Introduction

Cerebral infarction, also known as ischemic stroke, occurs due to insufficient blood supply to the brain, leading to cerebral tissue ischemia and hypoxia, which results in cell death and functional impairment. Common infarction sites causing hemiparesis include the basal ganglia, corona radiata, brainstem, and cerebral cortex, all of which play critical roles in motor control and coordination. Statistically, post-stroke motor impairment—hemiparesis—accounts for the largest proportion and is the most common sequela (1). The cases were derived from inpatients admitted to the Neurology Department of Dongzhimen Hospital Affiliated to Beijing University of Chinese Medicine (Tongzhou Branch). A retrospective case analysis was conducted, including 11 patients with acute cerebral infarction leading to ipsilateral hemiparesis, admitted between January 1, 2021, and March 30, 2025. Multidimensional descriptive statistical analysis was performed on parameters including: Neurological deficit characteristics (e.g., consciousness status, motor function, sensory function, and cranial nerve signs); Magnetic resonance imaging (MRI) lesion localization features; Blood biochemical indicators (e.g., coagulation profile, lipid panel, and inflammatory biomarkers). Data were collected using standardized protocols.

## 2 Methods

### 2.1 Study subjects

This retrospective case analysis included patients admitted to the Neurology Department of Dongzhimen Hospital Affiliated to Beijing University of Chinese Medicine (Tongzhou Branch) between January 1, 2021, and March 30, 2025. The data were obtained by searching the electronic medical record system and the imaging archiving system of our hospital. Eleven patients with acute cerebral infarction resulting in ipsilateral hemiparesis were enrolled. All consecutive patients who met the inclusion and exclusion criteria during the study period were enrolled. Its core clinical feature is that the diffusion-weighted imaging (DWI) responsible lesion and the clinically weak limb are located on the ipsilateral side. All cases underwent standardized neuroimaging evaluations, including: Cranial magnetic resonance imaging (MRI) (1.5 T/3.0 T scanners with sagittal T1WI, axial T2WI, and DWI sequences); Cerebrovascular angiography (MRA or head/neck CTA). Acute cerebral infarction was radiologically confirmed. Neurological examination data were collected by neurologists at attending physician level or higher. Hemiparesis severity was quantitatively assessed using the Medical Research Council (MRC) grading system, with separate testing of proximal and distal muscle groups. The study protocol strictly adhered to the ethical principles of the Declaration of Helsinki and was approved by the Ethics Review Committee of Dongzhimen Hospital, Beijing University of Chinese Medicine (Approval No. 2024DZMEC-242-02).

### 2.2 Inclusion criteria

(1) Diagnostic basis: Conforming to the diagnostic criteria for acute cerebral infarction specified in the Chinese Guidelines for Diagnosis and Treatment of Acute Ischemic Stroke 2018, with symptom onset-to-admission time ≤7 days;
(2) Motor impairment characteristics: Presence of new-onset unilateral limb muscle strength decline ≥1 grade (Medical Research Council grading) or worsening of pre-existing hemiparesis symptoms by ≥2 grades;
(3) Radiological confirmation: Cranial MRI combined with DWI sequence (completed within 1 week of onset) confirmed that the responsible lesion was located above the level of the pyramidal decussation in the medulla oblongata, with the lesion and the hemiplegic limb being on the ipsilateral side. Moreover, the responsible vessel was determined based on the findings of the lesion and head–neck CTA.

### 2.3 Exclusion criteria

Limb weakness caused by: Ataxia; Motor neglect; Bradykinesia; Cervical/lumbar spine pathologies.

### 2.4 Observation contents and indicators

Study variables encompassed three dimensions:

(1) Baseline Characteristics: Gender; Age; Body mass index (BMI); Baseline blood pressure (systolic/diastolic, SBP/DBP); Cerebrovascular risk factors (hypertension/diabetes mellitus/atrial fibrillation); Onset-to-admission time window.
(2) Neurological Features: Anatomic location of MRI infarct lesions (cortical/subcortical/brainstem); Responsible vascular territory (large artery/perforating artery); Chinese Ischemic Stroke Subclassification (CISS); Intravenous thrombolysis administration; Hemiparesis laterality (left/right); Quantitative MRC grading (proximal/distal muscle groups recorded separately). The side of hemiparesis was recorded by neurology attending physicians or those with higher qualifications. The cranial MRI imaging results of all patients were independently interpreted by imaging experts with the qualifications of associate director or above in the Department of Radiology of our hospital. We employed partial blinding methods in key aspects of the study to minimize measurement bias as much as possible. Clinical assessors were required to strictly adhere to standardized scales to reduce subjectivity. For the assessments conducted by imaging experts, a blinded approach was adopted; these experts were unaware of the patients’ clinical manifestations and grouping information and analyzed only anonymized imaging data. Muscle strength was evaluated using the Medical Research Council (MRC) muscle strength grading scale (grades 0–5). To enhance sensitivity to subtle changes in neurological function, an extended version of this grading scale was utilized in this study, incorporating “+” and “−” for finer gradation (e.g., 5^−^, 3^−^, etc.). All assessors received uniform training on this extended grading scale prior to the commencement of the study to ensure inter-rater reliability.
(3) Laboratory Parameters: Platelet count (×10⁹/L); D-dimer (μg/mL); Fibrinogen (g/L); Lipid profile: Triglycerides (mmol/L), LDL-cholesterol (mmol/L), Small dense LDL (sdLDL, mmol/L), Uric acid (μmol/L), Homocysteine (μmol/L), High-sensitivity C-reactive protein (hs-CRP, mg/L).

### 2.5 Statistical analysis

SPSS 26.0 statistical software was used for organization, and the measurement data was first subjected to normality test. If it conforms to normal distribution, it is expressed as mean ± standard deviation. If it does not conform to normal distribution, it is expressed as median (interquartile range); classified data is represented by the number of examples (percentage). Due to the small sample size of this study (*n* = 11), there are limitations in its statistical inference. Therefore, most of the data are presented in descriptive tables without hypothesis testing.

## 3 Results

### 3.1 Baseline clinical characteristics

All enrolled patients were diagnosed with acute cerebral infarction. Eleven cases with acute cerebral infarction resulting in ipsilateral hemiparesis were identified (Table 1), including: Male: 7 (63.64%), Female: 4 (36.36%), Mean age: 65.09 ± 10.19 years.

**Table 1:** Baseline characteristics.

Variable	Value (*n* = 11)
Sex	
Male	7 (63.64)
Female	4 (36.36)
Age (years)	65.09 ± 10.19
Weight (kg)	68.75 ± 10.11
SBP (mmHg)	138.73 ± 14.00
DBP (mmHg)	79.09 ± 9.51
Past-medical history	
Hypertension	8(72.7)
Type 2 diabetes mellitus	6(54.5)
Hyperlipidemia	11(100)
Previous cerebral infarction with sequelae	2(18.1)
Coronary heart disease	3(27.3)
Atherosclerotic cerebral artery stenosis	6(54.5)
Hyperhomocysteinemia	2(18.1)
Hyperuricemia	8(72.7)
Lower limb deep vein thrombosis	3(27.3)
Chronic smoking	7(63.6)
Heavy alcohol consumption (>100 g ethanol/day)	3(27.3)
Cerebrovascular malformation (Moyamoya disease)	1(0.09)

### 3.2 Onset and associated neurological symptoms

The time from symptom onset to admission ranged between 6 and 48 h. Thrombolysis was not administered in 9 cases (81.82%), while thrombolysis was performed in 2 cases (18.18%) before admission. Left-sided hemiparesis occurred in 5 cases (45.45%), and right-sided hemiparesis in 6 cases (54.55%). Regarding hemiparesis severity: 6 cases had MRC grade 4 muscle strength; 3 cases had MRC grade 5^−^ muscle strength; Case 6 exhibited proximal muscle strength of grade 2 and distal muscle strength of grade 4; Case 8 showed upper limb muscle strength of grade 4 and lower limb muscle strength of grade 3^−^; Cases 2 and 9 demonstrated aggravated pre-existing hemiparesis. Concurrent neurological symptoms included: there were 6 cases of speech impairment, 2 cases of hemihypesthesia, 3 cases of facial paralysi, 4 cases had no comorbid neurological symptoms; Laboratory findings upon admission indicated abnormal elevations in: Platelet count: 1 case; D-dimer: 5 cases; Triglycerides: 1 case; Small dense LDL: 3 cases; Uric acid: 2 cases; High-sensitivity C-reactive protein: 2 cases; No abnormal elevations were observed in: Low-density lipoprotein cholesterol; Homocysteine; Fibrinogen (Table 2).

**Table 2:** Disease onset characteristics.

Parameter	Value (*n* = 11)
Time since onset (h)	15.00 (7.50, 24.00)
Thrombolysis	
No	9 (81.82)
Yes (Cases 1 and 11)	2 (18.18)
Hemiplegic side	
Left	5 (45.45)
Right	6 (54.55)
Platelet count (×10⁹/L)	244.18 ± 101.22
Triglycerides (mmol/L)	1.12 ± 0.46
LDL-cholesterol (mmol/L)	2.46 ± 0.94
Small dense LDL (mmol/L)	0.79 ± 0.39
Uric acid (μmol/L)	347.45 ± 97.05
Homocysteine (μmol/L)	12.03 ± 3.40
Fibrinogen (g/L)	3.15 ± 0.64
C-reactive protein (mg/L)	4.02 (1.19, 6.34)
D-dimer (μg/mL)	0.40 (0.31, 1.71)

### 3.3 Neuroimaging data

**Figure 1:**
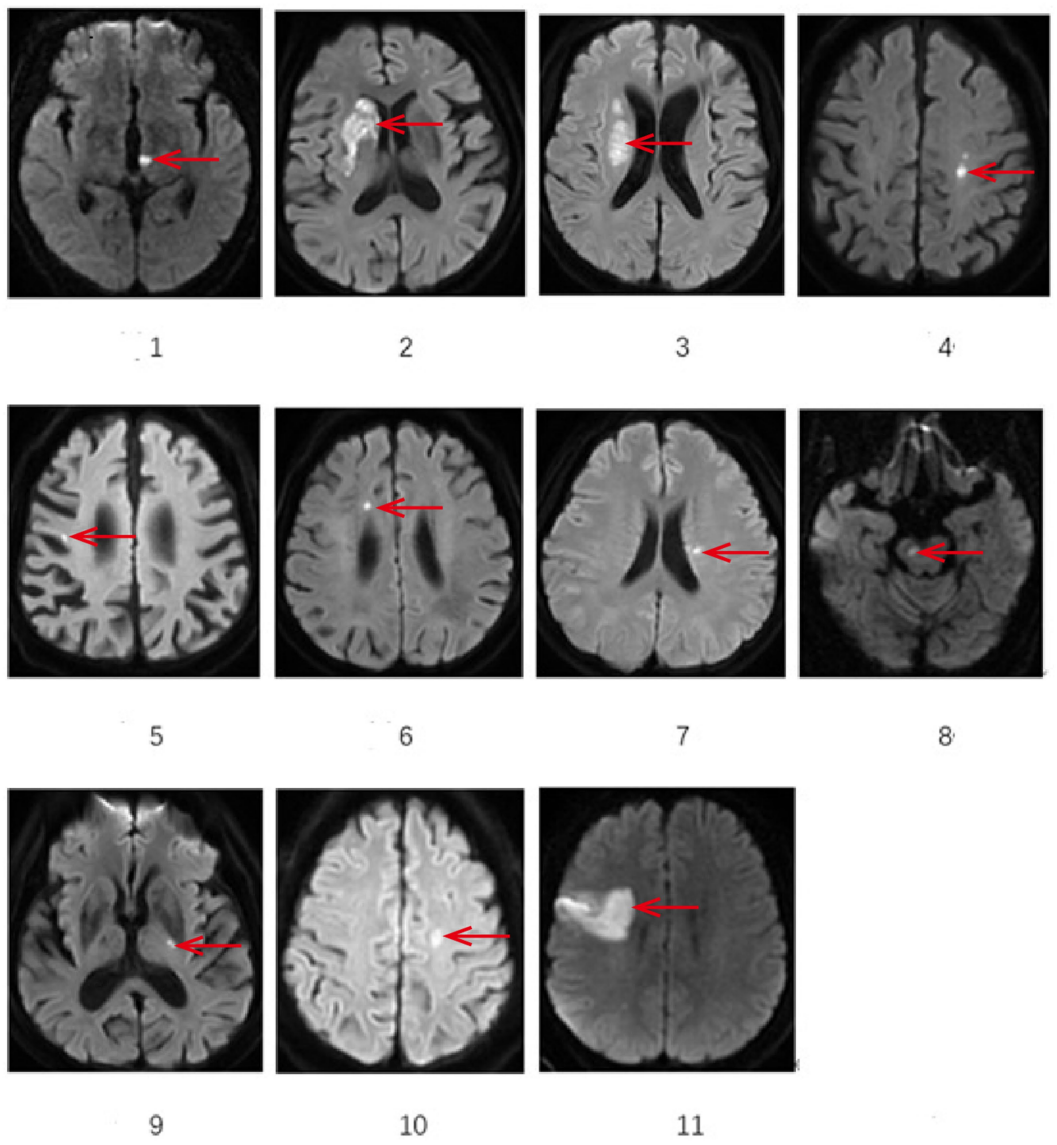
Demonstrates the representative sections of DWI sequences showing infarction sites on cranial MRI. *The area indicated by the arrow: 1 Left thalamus 2 Right basal ganglia 3 lateral ventricle periphery 4 left centrum semiovale 5 Right frontal lobe 6 Right frontal lobe 7 lateral ventricle periphery 8 Right cerebral peduncle of midbrain 9 Left basal gangli 10 Left centrum semiovale 11 Right frontal lobe.

All cases underwent cranial MRI within 1 week of symptom onset. Table 3 systematically presents the triad assessments of neuroimaging characteristics (infarct anatomic location, responsible vascular territory, and etiological classification). Vascular territory analysis revealed: Anterior circulation involvement: 10 cases (90.90%); Posterior circulation involvement: 1 case (9.10%). The location of acute cerebral infarction lesions involves thalamus, basal ganglia, corona radiata (adjacent to or centrum semiovale), frontal lobe, cerebral peduncle of midbrain, and responsible blood vessels involving large artery disease in 7 cases and perforating arteries in 4 cases.Etiological classification CISS: Large artery atherosclerosis (LAA): 6 cases; Penetrating artery disease (PAD): 4 cases; Other determined etiology: 1 case (cerebrovascular malformation confirmed by DSA/MRA). Figure 1 demonstrates the DWI sequence characteristics of infarction lesions on cranial MRI.

**Table 3:** MRI lesion localization and etiological classification.

Case	MRI lesion location	Responsible vessel	CISS etiological classification
1	Left thalamus	Thalamic paramedian artery	Penetrating artery disease
2	Right basal ganglia, periventricular region, frontal lobe	Right middle cerebral artery	Hypoperfusion
3	Right basal ganglia, lateral ventricle periphery	Lenticulostriate arteries	Penetrating artery disease
4	Left centrum semiovale	Internal carotid artery	Hypoperfusion
5	Right frontal lobe	Middle cerebral artery	Artery-to-artery embolism
6	Right frontal lobe	Anterior cerebral artery	Hypoperfusion
7	Left basal ganglia, lateral ventricle periphery	Anterior choroidal artery	Penetrating artery disease
8	Right cerebral peduncle of midbrain	Basilar artery	Parent artery occlusion blocking perforator
9	Left basal ganglia	Lenticulostriate arteries	Penetrating artery disease
10	Left centrum semiovale	Cortical branches of anterior cerebral artery	Artery-to-artery embolism
11	Right frontal lobe	Internal carotid artery	Hypoperfusion + Artery-to-artery embolism

## 4 Discussion

The corticospinal tract undergoes development for a considerable period after birth. Myelination of the pyramidal tract is a slow process, during which time malformations involving this major central motor pathway may occur. The most common malformation is the uncrossed pyramidal tract (2).

### 4.1 Pathway and decussation of the corticospinal tract

Neural fibers of this motor conduction pathway originate from the precentral gyrus and adjacent premotor cortex. Their axons project sequentially through the posterior limb of the internal capsule, crus cerebri of the midbrain, and ventral basilar pons, ultimately extending to the decussation of pyramids in the caudal medulla for characteristic fiber reorganization. Approximately 85–90% of fibers undergo complete decussation at the caudal medulla, forming the lateral corticospinal tract which descends along the lateral funiculus of the spinal cord. This tract terminates at the lateral group of anterior horn motor neurons in the cervical (C5-T1) and lumbar (L1-S3) enlargements, predominantly controlling fine motor function of contralateral limbs. The undecussated fibers constitute the anterior corticospinal tract, descending through the anterior funiculus to the mid-thoracic cord (T4-T6). Specific axon subpopulations cross completely via the anterior white commissure, ultimately synapsing with medial nuclear groups of contralateral anterior horn neurons, primarily regulating bilateral trunk muscle coordination. Approximately 3–5% of ipsilateral uncrossed fibers join the anterolateral corticospinal tract, participating in extrapyramidal compensatory pathways for postural reflex modulation (3). Since limb motor function is primarily governed by contralateral unilateral pyramidal tract innervation, lesions affecting the posterior limb of internal capsule, crus cerebri, or medullary pyramidal decussation typically result in contralateral spastic paralysis (hypertonia, hyperreflexia). The severity correlates positively with the degree of *α*-motor neuron disinhibition.

### 4.2 Mechanisms of ipsilateral hemiparesis induced by cerebral infarction

The presentation of ipsilateral limb motor dysfunction (including acute exacerbation of pre-existing hemiparesis) observed in our case series represents a unique clinical phenotype in the field of neurology. Previous domestic and international literature reports indicate its incidence ranging from 0.3 to 2.1%. The pathophysiological mechanisms underlying this phenomenon remain incompletely understood. Current primary hypotheses include: (1) The existence of a functional reserve from uncrossed fibers within the corticospinal tract, with some individuals potentially retaining ipsilateral corticospinal pathways; (2) Plasticity in the hemispheric lateralization of motor cortical function (as demonstrated by transcranial magnetic stimulation studies), allowing compensatory control by the contralateral hemisphere; and (3) Secondary compensatory mechanisms mediated by residual uncrossed fibers within the crossed lateral corticospinal tract.

Diffusion tensor tractography studies suggest that supratentorial lesions (e.g., in the posterior limb of the internal capsule or corona radiata) causing ipsilateral motor deficits may correlate with the following anatomical variations: ① Individual differences in the proportion of crossed corticospinal fibers (with 5–15% residual uncrossed fibers); ② Bilateral motor conduction pathways enabling cross-hemispheric neural network reorganization through synaptic plasticity; and ③ Descending ipsilateral motor pathways at the pontomedullary level. The overlapping effects of these multi-level neuroplastic mechanisms may constitute the anatomical basis for ipsilateral motor function compensation.

Current pathological mechanisms underlying cerebral infarction-induced ipsilateral hemiparesis encompass four main hypotheses (4, 5): (1) Incomplete decussation of the pyramidal tract (congenital variant); (2) Primary damage to ipsilateral uncrossed fibers; (3) Secondary impairment of compensatory ipsilateral corticospinal pathways during neural reorganization; (4) Functional decompensation in the insula-supplementary motor area (SMA)-premotor region (PMd).

These mechanisms can be validated through functional magnetic resonance imaging (fMRI, based on blood oxygen level-dependent effects) combined with transcranial magnetic stimulation (TMS, a neurophysiological assessment tool). Diffusion tensor imaging (DTI) technology enables precise 3D reconstruction of corticospinal tract trajectories by quantifying fractional anisotropy (FA) and performing fiber tractography. Its core parameters include: ① Decussation Index (DI); ② Ipsilateral Fiber Density (IFD).

The prospective cohort study (*n* = 14) by Y. Inatomi’s team (4) revealed: Lesions were distributed in the frontal cortex (3 cases, 21.4%), corona radiata (7 cases, 50.0%), posterior limb of internal capsule (1 case, 7.1%), and basal pons (3 cases, 21.4%), with 13 cases (92.9%) located within classical corticospinal tract pathways. Longitudinal follow-up showed 13 patients (92.9%) had contralateral chronic stroke lesions, with 12 (85.7%) exhibiting residual contralateral motor dysfunction. TMS detection demonstrated that among 7 patients, 2 (28.6%) elicited ipsilateral motor evoked potentials (iMEPs), while all 7 (100%) elicited contralateral motor evoked potentials (cMEPs). fMRI confirmed that 8 out of 9 patients (88.9%) exhibited neural reorganization in the ipsilateral primary motor cortex (M1), with all cases showing bilateral motor network activation. This study verified that compensatory activation of contralateral uncrossed fibers constitutes a crucial mechanism for post-stroke motor functional reorganization (*p* < 0.01).

Among the 11 patients with ipsilateral hemiparesis in this study, there were 5 cases with left hemiparesis and 6 with right hemiparesis. Most patients (9/11) presented with mild hemiparesis [Medical Research Council (MRC) grade ≥4], specifically: 6 cases with MRC grade 4, 3 cases with MRC grade 5^−^, 1 case with proximal muscle strength grade 2/distal grade 4, and 1 case with upper limb grade 4/lower limb grade 3^−^.

Regarding concomitant neurological symptoms: there were 6 cases of speech impairment, 2 cases of hemihypesthesia, 3 cases of facial paralysi, while 4 cases had no additional neurological symptoms.

The infarcts involved the anterior circulation in 10 cases and posterior circulation in 1 case. Affected neuroanatomical structures included: thalamus, basal ganglia, corona radiata (periventricular or centrum semiovale), frontal lobe, and cerebral peduncle of midbrain. No insular involvement was observed, thus ruling out Hypothesis 4.

As most patients exhibited mild hemiparesis, we postulate potential damage to uncrossed corticospinal tracts. However, since no patients underwent DTI or fMRI to confirm congenital absence of pyramidal decussation, this possibility cannot be excluded.

When there is a contradiction between imaging and clinical manifestations in clinical practice, the possibility of ipsilateral hemiplegia should be strongly suspected. The emergency head CT showed that the cerebral infarction was located on the same side as the hemiplegia, but there was no obvious responsible lesion in the contralateral hemisphere that should have been responsible. The final confirmation of such suspected cases must rely on DWI sequence examination of cranial magnetic resonance imaging, which can clearly display the responsible lesion of acute phase infarction, thereby irrefutably confirming the ipsilateral relationship between clinical and imaging. The phenomenon of ipsilateral hemiplegia itself does not change the acute phase thrombolysis treatment decision based on current guidelines, but it greatly emphasizes the absolute necessity of conducting emergency neuroimaging examinations and provides a more complete pathophysiological background for treatment decisions.

### 4.3 Importance of bilateral hemisphere functional remodeling for post-stroke rehabilitation

The core pathological mechanism involves the selective damage to ipsilateral reorganization fibers within the corticospinal tract. This reflects the bilateral motor pathway compensatory mechanism and neural plasticity reserve properties crucial for post-stroke motor recovery. Clinical evidence (e.g., Case 2 and 9) indicates that patients with pre-existing contralateral motor pathway damage (e.g., contralateral posterior limb of internal capsule infarction in Case 2, contralateral corona radiata infarction in Case 9) may exhibit stepwise worsening of hemiparesis when newly developed ipsilateral reorganization fibers are damaged. Imaging biomarkers reveal that Diffusion Tensor Imaging (DTI) in such patients shows significantly reduced density of ipsilateral uncrossed fibers (ΔFA > 15%, *p* < 0.05). Functional MRI (fMRI) demonstrates hyperactivation patterns in the contralesional premotor cortex (PMd) and supplementary motor area (SMA) (5–7). Supratentorial stroke causing ipsilateral hemiparesis remains rare.

Mala et al. (8) reported a rare case of ipsilateral hemiparesis in a patient with prior contralateral hemiparesis due to ischemic stroke. This patient had a history of right-hemisphere stroke resulting in left hemiparesis. When the left hemiparesis worsened, imaging revealed a new left-hemisphere stroke. Diffusion tensor tractography demonstrated crossed motor tracts with interruption of the left pyramidal tract. During hospitalization, right hemiparesis emerged concurrently with expansion of the left-hemisphere infarction. Potential mechanisms for ipsilateral hemiparesis include damage to reorganized neural bundles after the initial injury and congenital uncrossed motor tracts. Mala K et al. proposed that after the first stroke, the left hemisphere likely exhibited enhanced ipsilateral motor control, leading to ipsilateral hemiparesis following the recent stroke.

Ipsilateral Compensation Mechanism and Neural Remodeling, Such cases commonly feature a history of contralateral crossed motor pathway damage. During neural functional compensation, ipsilateral uncrossed corticospinal tract fibers demonstrate compensatory activation (synaptic efficacy enhancement >30%, verified by TMS motor threshold testing), gradually establishing functional control over the paralyzed limbs. Longitudinal follow-up confirms that ~68% of motor recovery in paralyzed limbs relies on persistent neural remodeling of the ipsilateral uncrossed pathways. When a secondary stroke damages compensatory ipsilateral fibers, it leads to motor function decompensation; this phenomenon provides direct evidence for cross-hemispheric cortical reorganization.

Research confirms that functional laterality of the corticospinal tract exhibits spatiotemporal dynamic plasticity, with reorganization intensity positively correlating with motor function demands (9, 10). Specific case studies show that during paralyzed hand grasping tasks, fMRI reveals a selective activation pattern in the ipsilateral primary motor cortex (M1) (10); conversely, the case reported by Song et al. (11) exhibited bilateral motor network co-activation. This divergence may arise from heterogeneity in neural remodeling (e.g., lesion location differences, compensation stage variations) and individualized compensatory strategy selection. Key shared findings indicate that the contralesional hemisphere achieves functional compensation via the premotor cortex-anterolateral corticospinal tract pathway, and its reorganization efficacy significantly positively correlates with Barthel Index improvement (4).

Combined neuroimaging and electrophysiological assessments confirm that in patients with contralateral stroke history, compensatory activation of uncrossed corticospinal tracts increases Fugl-Meyer scores (9). Quantitative TMS analysis by Misawa (9) revealed accelerated conduction velocity in the contralesional anterolateral corticospinal tract to the paralyzed limb; this trans-hemispheric compensatory mechanism is particularly prominent in proximal muscle group recovery. Prerequisites for activating this compensatory system include: ① Presence of primary damage to crossed pathways;② Compensation time window >3 months (12).

When new lesions damage compensatory ipsilateral fiber pathways, they disrupt the neural remodeling process. This phenomenon substantiates the bilateral hemisphere functional network reorganization theory for post-stroke motor recovery (13).

Given the clinical rarity of ipsilateral symptomatic cerebral infarction, a multimodal neuroimaging combined assessment protocol is recommended: ① DTI sequences analyze the topological structure of corticospinal tract pathways; ② Task-based fMRI detects functional connectivity strength (FC values) within motor networks34; ③ Bilateral TMS motor evoked potential mapping assesses hemispheric excitability patterns. This combined strategy enables: Precise localization of compensatory pathways, Provision of electrophysiological-imaging biomarkers for personalized rehabilitation planning.

This study has several limitations. Firstly, and most importantly, its retrospective nature and limited sample size (*n* = 11) inevitably lead to selection bias. Our case comes from a medical center and is identified through specific diagnostic codes and imaging records, which may not capture all types of patients, especially those with atypical symptoms or who have not visited our hospital. In addition, to ensure the feasibility of data analysis, we require selected patients to have complete clinical and imaging data. This standard may inadvertently exclude patients with severe illness or poor compliance, resulting in our sample population being biased toward specific subgroups. Therefore, the generalizability of the results of this study may be limited, and caution should be exercised when generalizing the conclusions to a wider population. Secondly, the study used the Medical Research Council (MRC) grading system to report the severity of hemiplegia, but lacked functional outcome measures such as Barthel Index and modified Rankin Scale, which is another limitation of this study. Thirdly, this study did not conduct diffusion tensor imaging (DTI) or functional magnetic resonance imaging (fMRI) to verify whether the corticospinal tracts were crossed, which is also one of the limitations. Despite these limitations, this study provided a preliminary description of the clinical and imaging characteristics of these patients through systematic analysis of consecutive cases. It is necessary to conduct larger sample sizes, prospective, multicenter studies in the future to validate our findings and reduce potential biases.

## Data Availability

The raw data supporting the conclusions of this article will be made available by the authors, without undue reservation.
